# Breast cancer screening in developing countries

**DOI:** 10.6061/clinics/2017(04)09

**Published:** 2017-04

**Authors:** René Aloísio da Costa Vieira, Gabriele Biller, Gilberto Uemura, Carlos Alberto Ruiz, Maria Paula Curado

**Affiliations:** I Programa de Pós-Graduação em Oncologia, Hospital de Câncer de Barretos, Barretos, SP, BR; II Programa de Pós-graduação em Obstetricia, Ginecologia e Mastologia, Faculdade de Medicina de Botucatu – UNESP, Botucatu, SP, BR; III Departamento de Obstetricia e Ginecologia, Faculdade de Medicina, Universidade de Sao Paulo, Sao Paulo, SP, BR; IV International Prevention Research Institute, Lyon, France

**Keywords:** Breast Neoplasms, Epidemiology, Prevention & Control, Mammography, Mass Screening, Developing Country

## Abstract

Developing countries have limited healthcare resources and use different strategies to diagnose breast cancer. Most of the population depends on the public healthcare system, which affects the diagnosis of the tumor. Thus, the indicators observed in developed countries cannot be directly compared with those observed in developing countries because the healthcare infrastructures in developing countries are deficient. The aim of this study was to evaluate breast cancer screening strategies and indicators in developing countries.

A systematic review and the Population, Intervention, Comparison, Outcomes, Timing, and Setting methodology were performed to identify possible indicators of presentation at diagnosis and the methodologies used in developing countries. We searched PubMed for the terms “Breast Cancer” or “Breast Cancer Screening” and “Developing Country” or “Developing Countries”.

In all, 1,149 articles were identified. Of these articles, 45 full articles were selected, which allowed us to identify indicators related to epidemiology, diagnostic intervention (diagnostic strategy, diagnostic infrastructure, percentage of women undergoing mammography), quality of intervention (presentation of symptoms at diagnosis, time to diagnosis, early stage disease), comparisons (trend curves, subpopulations at risk) and survival among different countries.

The identification of these indicators will improve the reporting of methodologies used in developing countries and will allow us to evaluate improvements in public health related to breast cancer.

## INTRODUCTION

Breast cancer is a global problem, and 1.7 million new cases are diagnosed per year. Approximately 60% of deaths due to breast cancer occur in developing countries (DCs) 1, whereas in the United States (US), an estimated 249,260 new cases of breast cancer are diagnosed each year, and mortality due to this disease is decreasing 2. In contrast, breast cancer in DCs represents one-half of all breast cancer cases and 62% of the deaths 1. In Brazil, it is expected that the incidence of breast cancer will be about one-fourth that of the US, but this increased incidence is associated with an increase in breast cancer-associated mortality 3.

Despite advances in medicine, breast cancer is diagnosed in the advanced stages in countries with limited resources because early detection, diagnosis, and treatment cannot be efficiently promoted. To evaluate the complexity of the healthcare system in relation to breast cancer, the “Breast Health Global Initiative” (BHGI) 4 has sought to categorize the organizational levels of countries in relation to breast cancer. Specifically, at the basic level, breast self-examination is encouraged, whereas diagnostic ultrasound and mammography are available at a limited level. At the increased level, patients have access to diagnostic mammography with opportunistic breast screening, and at the maximum level, the population undergoes organized screening for breast cancer 4.

In the US, 70% of women undergo mammographies. This percentage is higher in white women (72.1%), women with a higher level of education (80.1%), women born in the USA (71.6%), and women with health insurance (73.6%) 5. In countries with budgetary limits, the percentage of cancer diagnosis in the presence of a palpable mass 6 and adherence strategies based on self-breast examination are being discussed 7.

The prognosis for breast cancer is considered good. The survival rate is on the order of 73% in developed countries and 57% in DCs. In the US, the 5-year survival rate is 89.7% 8, 9. Although the incidence of breast cancer is lower in DCs, the mortality/incidence ratio is higher 8. Due to economic and logistical constraints, a limited organized network is aimed at the early diagnosis of breast cancer in DCs. Mammographic screening is not a reality, which is reflected in the high number of patients diagnosed at an advanced stage.

It is difficult to compare or evaluate the health systems of DCs, and it is also difficult to evaluate their improvement. Based on this condition, it is important to consider indicators that indirectly reflect the status and evolution of public health systems related to breast cancer screening and diagnosis. It is a challenge to identify possible indicators associated with the diagnosis of early breast cancer because these populations lack real indirect indicators related to breast cancer screening and indicators that can evaluate progressive improvements to the healthcare system or that can compare healthcare systems among DCs. The identification of these indicators and the subsequent comparisons are the purpose of this study.

## MATERIALS AND METHODS

This study consists of an integrative systematic review based on a systematic search methodology to evaluate possible indicators related to methods of breast cancer screening and diagnosis in DCs. According to the Brazilian National Ethics Committee resolution 466, systematic reviews do not need to be evaluated by an Ethics Committee. Study methodologies were not evaluated, but rather, publications that discussed this subject were examined.

The PubMed database was searched using the keywords “Breast Cancer” or “Breast Cancer Screening” and “Developing Country” or “Developing Countries”. This search returned 1,149 articles published between April 1974 to September 30th, 2015.

**Figure 1:**
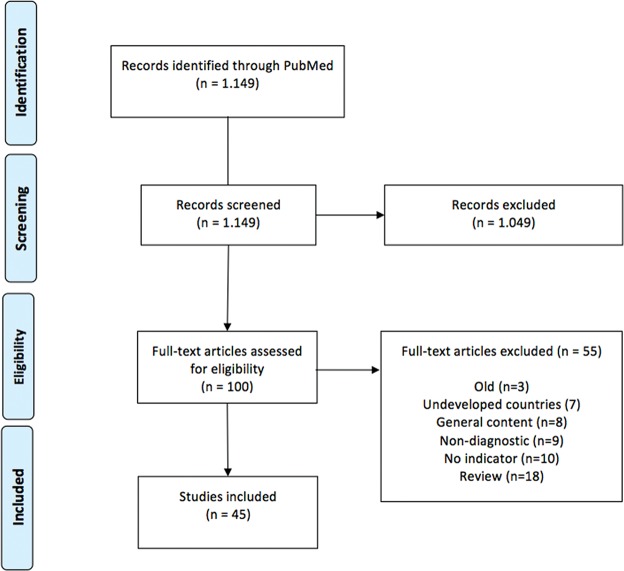
PRISMA records flow diagram.

After reading the titles and abstracts, we identified 100 potential articles discussing breast cancer indicators and screening characteristics. All 100 identified articles were read in full by two researchers (GB and RACV). Eighteen articles were excluded because they were review articles on screening strategies in DCs; 7 articles were excluded because they exclusively addressed underdeveloped countries; 3 articles were excluded because they were published in the 1990s and were superseded by subsequent publications with different data; 27 articles were excluded for various reasons. Thus, the 45 articles that were reviewed included numerical data regarding diagnostic methodology and tumor presentation at diagnosis. We included observational studies, prevalence studies and prevalence review articles. A summary of the results is shown in Figure 1.

We attempted to use the systematic review elements formatting structure, PICOTS (Population- Intervention- Comparator- Outcome- Timing- Setting) 10, 11, associating elements suggested by the BHGI for DCs 4, and quality criteria to be used in mammographic screening 12. A Microsoft Excel® table was generated, which identified the PMID, first author, main results and potential indicators based on the PICOTS structure. We then attempted to group studies according to subject matter, taking into account items that reflected a possible health indicator, the methodology used for breast cancer diagnosis, or items that reflected the characteristics of breast cancer patients in DCs (Table 1).

The results observed for each item were then described to better understand the characteristics of mammographic screening and possible outcomes observed in DCs.

## RESULTS

Based on PICOTS, where the scenario (Setting) is breast cancer diagnosis in DCs, a lack of controlled studies (Study design) was observed. We identified 45 articles consisting of 7 reviews and 38 original articles. These articles are presented in Table 1. In the intervention factor (I), we observed factors relating to diagnosis and factors relating to diagnostic quality. We present the individual results of each factor.

### Population

The incidence per 100,000 people differed, ranging from 9.5 in Nigeria 13 to 65 in the Fuji islands 14 and 92.2 in French Polynesia 14. Regional variations were also observed, but the incidence was generally lower in DCs than in developed countries 13, 14. Noteworthy regional reviews are available for Arab countries 13, 15, 16, Asia 14, 17 and Latin America 18.

### Intervention/Diagnosis

#### Diagnostic strategy methodology/guidelines

In many centers, breast self-examination (BSE) 19 and clinical breast examination (CBE) are keys to diagnosis when mammographic diagnosis is not feasible 19, 20, but many women are unaware of BSE and CBE 21. Furthermore, difficulties in promoting education related to BSE have been cited 15, with approximately 3% 22 to 24% of women administering a BSE 21, 23 and 12.5% undergoing a CBE 15.

In countries lacking government recommendations regarding mammography, the recommended starting age for routine mammography varies widely, with starting ages of 25, 40, or 50 years of age and upper limits of 64, 70, or 75 years of age; both annual or biennial repetition are suggested 16, 18,24-27.

#### Diagnostic infrastructure

A total of 5% of all worldwide expenditure on breast cancer screening takes place in DCs. This limitation in resources and the many competing priorities mean that conducting mammography and providing effective treatment are difficult 28 and that most tumors are consequently diagnosed at the advanced stages. Because breast cancer is often a fatal disease in some countries 29, screening and treatment are not considered cost-effective 30. In addition, many countries lack a national program 31-33 because national protocols regarding the appropriate age range for mammography are not available 34. These conditions lead to a lack of information about the importance of mammography. Similarly, BSE and CBE can be used as diagnostic and screening methods 35, but this strategy may not be employed at the public health level. In Nigeria, 75.6% of studied women had never performed a BSE, and only 58.2% had heard of BSEs 23. In Bangladesh, 41% of women did not know what breast cancer is, 71% did not know what screening is, and 96% did not know what a BSE is 36.

Some studies cite referral services that attenuate these problems and the use of breast ultrasound as an alternative method for the diagnosis of palpable lesions 37. One study describes four mammogram machines serving 7 million women 37, and another states that only working women had access to mammography 38. In fact, regions lacking public mammography service have also been described 39. Therefore, diagnostic mammography rates are approximately 0.5% 33, 40.

Other reports describe slightly better situations, including diagnostic mammography, population campaigns, and opportunistic mammography. A study from Jordan reports 14 mammography centers, with 7% of the population having mammograms and 17.9% having undergone screening 26. In Mexico, 22% 24 of the population receives regular mammograms. Positive educational interventions 41, isolated regional experiences of the first round of mammographic screening 27, 42, and structured locations where only a minority of patients are diagnosed late 43, 44 have been reported.

#### Percentage of women undergoing mammography

A study conducted in Jamaica reported that 11.4% of women had undergone a mammography at least 5 years ago 45.

In areas where mammography is opportunistically available, 42.1% of women aged 40-69 years had never had a mammogram 42, and these women are usually less educated and of a lower socioeconomic class 46.

### Intervention/quality

#### Presentation of symptoms at diagnosis

In the absence of screening strategies, clinical examination leads to diagnosis, and palpable tumors have been reported to represent 90 to 100% of all cases at diagnosis 33, 36, 44, 47. This proportion decreases to 26% after opportunistic mammographic screening 48.

The size of invasive tumors can also be used to evaluate disease, and the average sizes in Singapore and Malaysia are 2.2 cm and 3.0 cm, respectively 49. A review evaluating tumor characteristics in Asia and Africa reveals tumors averaging 3.3 cm in Tunisia, and 4.8 cm in Sudan and Nigeria. In Pakistan, 80% of tumors are larger than 2 cm 50, 51.

#### Time between the finding of a breast abnormality or examination and diagnosis

Because of both technology-based inefficiency and problems with access, few patients undergo treatment in well-equipped centers that feature protocols. Limited technology, economic reasons, and nutritional problems lead to maintenance of the disease's prevalence 20. Furthermore, this equipment may be available but in insufficient numbers to serve the entire population quickly. Therefore, a long time can elapse between a clinical complaint and the start of treatment. In better structured areas, limited equipment and diagnostic flow in the public network leads to delays in diagnosis, which reportedly range from 1 to 3 52, 8 53 and 10 months 33. These delays are inversely proportional to the degree of organization of the healthcare system 54. Generally, the delay is less than 1 month in developed countries and more than 2.5 months in DCs 53.

#### Percentage of cases diagnosed at an early stage

In general, the rate of patients diagnosed with carcinoma *in situ* is low, ranging from 1% in India 17 and Pakistan 55 to 7.4% in Iran 56. Considering early stage as clinical stage (CS) 0 and I, the rate of individuals diagnosed with early-stage tumors varies and is 5% in India 17, 10% in Pakistan 55, and 13.9% in Iran 56. In Brazil, the introduction of a regional screening program resulted in an increase in tumors diagnosed during the early stage from 14.5% to 43.2% 42. The infrastructure of Hong Kong differs and resulted in detection rates of 13% for CS 0 and 47% for early-stage disease 17. A study conducted in Malaysia compared women selected with mammographic screening and diagnostic mammography and found respective CS 0 diagnosis rates of 23.0% and 2.6% and respective early-stage cancer diagnosis rates of 53.8% and 27.0% 48.

The localized/regional/metastatic staging methodology is not used as frequently in publications in DCs, but we observed differences in diagnosis rates between DCs and developed countries 53, 57. Localized disease represents 25.2% of the tumors in Egypt, 27.8% in Saudi Arabia, 52.0% in Germany and 62.3% in the US 53.

**Figure 2:**
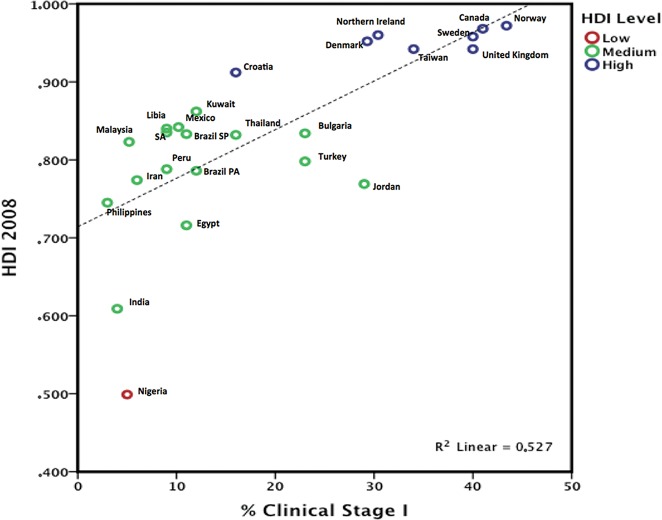
Scatter plot comparing the Human Development Index and the percentage of clinical stage I cases selected by country. PA = Porto Alegre; SA = Saudi Arabia; SP = São Paulo.

The TNM staging system is more often used in articles related to DCs. When only invasive disease is evaluated, stage I tumor staging rates were below 5% in India, the Philippines, and Nigeria, but the rates exceeded 30% in South Korea and Taiwan 50, 51, 53. Based on three reviews 50, 51, 53, we compared the percentage of clinical stage I patients and data from the Human Development Index 13, 58; we chose the year 2008 as a reference because that was the publication year of the articles reviewed. Only one publication was selected by country, and we chose only countries with an estimated HDI. Data were entered and plotted using IBM SPSS Statistics 20 for Mac® (Armonk, New York, NY). We observed a linear relationship (R^2^= 0,526) related to HDI and clinical stage, as a high HDI was associated with a higher rate of clinical stage I disease, and a lower HDI was associated with lower rate (Figure 2).

### Comparisons

#### Trend curves/temporal data

Studies demonstrating temporal changes indirectly show changes in the healthcare system. For example, a study in Egypt showed that the localized and metastatic disease rates were 14.8% and 14.0% in 1999, respectively, whereas they were 20.8% and 11.9%, respectively, in 2008. This change may be attributed to improvements in the local healthcare system 59. Similarly, a study of a Lebanese hospital showed a small improvement in the early diagnosis rate (CS 0 + I), which was 23.8% from 1990-1995 but changed to 25.8% from 2008-2013 25. A study conducted in Iran did not show temporal changes in the early-stage diagnoses between 1994-1997 and 2006-2009, which were 13% and 13.9%, respectively 56.

#### Subpopulations in the same country

A study conducted in Iran compared women of different socioeconomic classes; the BSE, CBE and mammography rates were 4.1%, 5.6% and 4.8%, respectively, among the poorest women and 83.9%, 90.3% and 87.4%, respectively, among women of a higher socioeconomic class 60. Similarly, black people in South Africa had lower rates of localized staging (5%) at diagnosis compared with whites (31%), a fact that reflects socioeconomic differences relating to diagnostic access 57, 61.

#### Comparisons among countries

**Figure 3:**
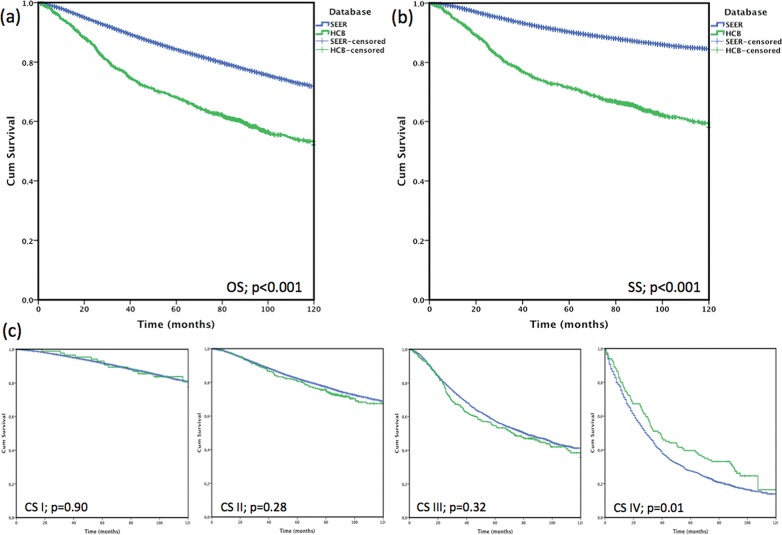
Survival according to the SEER study (blue) and a Brazilian Oncologic Hospital (HCB, green). (a) Overall survival (OS); (b) cancer-specific survival (SS); (c) overall survival selected by clinical stage (CS) at diagnosis. Unpublished Figure (ref 62) authorized by the Authors.

Because of limitations of organized databases in DCs, studies on this subject are also limited. In Brazil, comparing a hospital records series with Surveillance, Epidemiology and End Results (SEER) data, Brazilian patients were younger, demonstrated a longer medical history, presented with larger tumors and were less likely to have clinical stage I disease (10.2% x 50.1%), which were all reflected by the lower overall survival rate of 19.6 percentage points and a cancer specific survival of 26.9 percentage points. This effect almost disappeared when comparing overall survival by stage [Figure 3 62], which suggests that the difference may have been due to the excessive number of patients diagnosed at the early stage in the US 46.

#### Outcome/Survival

Despite the lower incidence rates, 45% of worldwide breast cancer cases and 54% of deaths due to breast cancer worldwide occur in DCs 63. The mortality/incidence ratio reflects the structure of these countries better than simple numeric mortality data, and this ratio exceeds 50% in many countries 14, 18, 64. Moreover, the per capita income directly correlates with incidence and inversely correlates with mortality 65, reflecting late diagnoses, poor healthcare and high mortality 66 in DCs. In many countries, incidence 14 and mortality tended to increase 1, 18, 67, whereas mortality tended to decrease in high-income countries 67.

Moreover, the survival rate was 11% higher in Singapore than in Malaysia. However, differences in tumor size at diagnosis, clinical stage at diagnosis, and treatment were also observed, all of which contributed to a higher survival rate of Singaporean patients 49.

## DISCUSSION

This study is a literature review that describes potential indicators related to the diagnosis of breast cancer in developing countries. Based on this study type, we opted to use the methodology of systematic reviews, using PRISMA and PICOTS, to evaluate possible ways that the health systems in DCs can be assessed. It was not possible to perform a meta-analysis with odds ratios and forest plots because a meta-analysis requires case-control or randomized studies, which are not often published in DCs. Because we found observational studies, prevalence studies and prevalence review articles, we opted to perform an integrative systematic review. The studies were summarized by topic according to the PRISMA criteria for selection. We identified potential indicators (Table 1) that can be used to compare differences and to evaluate improvement in public health systems.

BSEs and CBEs have long been considered to be important methodologies to be implemented at the population level. However, scientific evidence regarding the effectiveness of CBEs or BSEs in reducing mortality from breast cancer is currently lacking. Randomized studies carried out in China 68 and Russia 69 have led to a loss in support for BSEs as an early detection method because the breast cancer mortality rates did not differ between women who performed BSEs and those who did not. In this context, women are encouraged to be alert to any changes in the breast, and CBEs are part of this awareness and may lead the woman to a diagnostic evaluation. According to the Brazilian Society for Mastology, BSEs/CBEs in isolation are not encouraged but are always associated with the use of mammography, especially in women over 40.

The sensitivity of breast self-examination (BSE) is low (20 to 30%), and it is not associated with a decrease in mortality. Mammography has a sensitivity of 63 to 95% 37, and it is the only exam that has been demonstrated to be related to a decrease in mortality. Its sensitivity increases with the presence of palpable lumps and decreases in dense breasts. Approximately 10%-30% of breast cancers are not detected by mammography because of many possible factors such as dense parenchyma, obscure lesions, poor positioning, perception error, incorrect interpretations, subtype tumors, slow growing tumors, the presence of interval carcinomas and smaller-sized tumors 70. Based on these factors, the woman must perform a regular evaluation by BSE and/or undergo a clinical breast evaluation by a healthcare professional. Together, these exams must be considered in the diagnostic evaluation in addition to a breast ultrasound in symptomatic women 71.

Breast cancer screening by mammography is the best secondary prevention methodology for the population; it serves as a disease intervention measure and promotes early detection in the asymptomatic phase. Specifically, screening substantially reduces the morbidity and mortality due to late diagnosis. The HIP study (Health Insurance Plan) was the first to demonstrate a reduction in breast cancer mortality (30%) as a result of mammographic screening. In the 2006 and 2009 Cochrane reviews, the reduction was approximately 15%. In the last review (2013), which only evaluated randomized studies, they did not observe a reduction in mortality, but a reduction on the order of 25% was found when observational studies were included; and when grouping all studies together, this reduction was 19% 72. The greater likelihood of reducing breast cancer mortality in several developed countries can be attributed to screening programs and the evolution of adjunct therapy 73, 74. More recent studies have questioned these figures, arguing that this reduction is on the order of 8%, but these studies were conducted in developed countries and many methodological problems were questioned 75.

Screening primarily reduces mortality in women between 50 and 69-74 years of age, with less dramatic results in women who are 40-49 years of age. Thus, mammography should be performed on a large scale at the population level for this age group to reduce breast cancer mortality. The Brazilian Society of Mastology suggests that the starting age should be 40 years 76, which was also suggested by the American Cancer Society until 2015 77. In 2016, this suggestion was changed to age 45 78. EUSOMA 12, the US Preventive Services Task Force 79 and the Brazilian Ministry of Health 42 suggest that organized population screening commence at age 50.

Currently, many studies arguing against and in favor of mammographic screening are available. Decreases in the size of diagnosed lesions, decreases in breast cancer mortality, longer lifespans, an acceptable overdiagnosis level (1-10%), and the frequency of carcinoma *in situ* all support the use of mammographic screening 12, 75, 80. Conversely, partial evaluations of systematic reviews 80, discussions about the actual decline in advanced stage tumors in the US 81, and the rate of overdiagnosis (31%) 81 do not support the use of mammographic screening. Moreover, although some studies show that lives are indeed saved by mammographic screening, the number of survivors is low 82, 83. In general, studies of doctors who see patients support the use of screening 75, 77, whereas studies performed by epidemiologists argue against this type of screening 81, and suggest that women should be well informed regarding mammographic screening and aware of the pros and cons 82, 83. This fact is especially relevant in developed countries, where high income and education levels characterize much of the population.

In countries with extremely limited public health resources, infectious diseases are the main public health problem. The extent to which these diseases are controlled to increase life expectancy changes disease profiles. The age distribution of the population affects the incidence of cancer, with a decrease in age at diagnosis being observed in some countries 37. In DCs, most of the population earns a low income. Therefore, healthcare usually depends on government actions and public health infrastructure. Healthcare provision is related to the availability of resources, and many diseases compete for these resources. Public health practices are linked to national guidelines and available methodologies, which are associated with public education processes and the availability of public network demand absorbency. Therefore, not only tumor-related or epidemiological criteria but also account resource availability are important when evaluating the age range for screening strategies because data in DCs are generally limited and rely on studies conducted in developed countries. Thus, strategies are lacking, and BSEs are used as a screening strategy, whereas ultrasound is used as a diagnostic strategy.

In DCs, mammograms are not performed primarily because of barriers in the healthcare system, which are affected by the following: the accessibility of health services, unsatisfactory medical adherence due to public healthcare system limitations, the cost of tests, and difficulty in implementing follow-up tests 5, 84. The evaluation of factors relating to the healthcare system and non-adherence to mammogram screening guidelines is complex because such evaluations lack a specific indicator. The cultural context is interwoven with the infrastructure, the limitation of trained personnel, and the effective stratification of examinations up to the point of diagnosis, which should be quick, comprehensive, and effective. Nevertheless, this effect can be assessed by evaluating mammography available to the public, population coverage, and the percentage of mammography exams performed. In this context, access to mammography refers to the presence of this technology, the ease of the general population's access to it, the quality of the tests performed, and the possibility of performing additional tests focused on biopsy and differential diagnosis. Logistical and technological limitations delay tests prior to diagnosis, result in low population coverage, and generally limit access to regular mammograms. DCs report difficulties with respect to mammography screening in women, while developed countries discuss the practice of mammography for specific ages, as 57.2% of the women aged 50 to 74 in the US 85 undergo regular mammography. In addition, the qualities of breast cancer screening methods and accreditation programs are reported in developed countries 12, 86, 87. This fact reflects the clinical stage at diagnosis, as early clinical stage disease (EC 0 and I) represents 5% of all tumors in India 17 and 50.9% in Europe 86. In DCs, tumors are usually palpable, large, and not at an early clinical stage at the time of diagnosis (Figure 2). Conversely, tumors diagnosed only by mammography are infrequent, and the incidence of carcinoma in situ is low. For example, carcinoma *in situ* varies and was 0.1% in African registries, 1% in India, 11.4% in Europe, 16% in North America and approximately 4.5% in other regions of the world 9, 17, 86.

Although tumors smaller than 2 cm can be detected by clinical examination, they must be superficial, and the great majority of these tumors that are detected by mammography are smaller than 2 cm 88. Technology is associated with a local infrastructure and is based on mammograms, ultrasound, biopsy (open or core biopsy) and pathology. This reflects the lower percentage of early breast cancers detected in DCs. The HDI shows the association of PIB per capita and the life expectancy, which may indirectly reflect the health system. People have a socioeconomic dependence on public health systems, and therefore, the public health system is not a choice but may be the only option for many women. To evaluate the clinical stage at diagnosis, the TNM staging system is a standard and acceptable approach. We observed different frequencies of stages based on clinical stage 0 to IV (five categories) or clinical stage I to IV (four categories). The evaluation of the percentage of cases at clinical stage 0 would be based primarily on mammography-detected tumors, but these data were not reported in all the included studies. If we use the four categories listed above, we must pay attention to clinical stage I. Tumors larger than 2 cm are frequently palpable and clinically detectable. Based on this observation, we generated Figure 2 and chose data from clinical stage I to IV, as data related to clinical stage 0 were insufficient. We observed a linear relationship of the HDI and clinical stage, as a high HDI was associated with a higher rate of clinical stage I, while a lower HDI was associated with lower clinical stage I (Figure 2).

In terms of survival, patients with early-stage disease exhibit excellent survival rates, whereas patients with metastasis at the time of diagnosis have limited survival. Differences in survival are primarily due to differences in clinical stage at the time of diagnosis and the ability to provide adjunct therapy at the population level, which is reflected in the 5-year survival rates in different countries 89. The CONCORD study evaluated population-based cancer registries from 2005-2009 and estimated the 5-year survival rate after observing lower survival rates in South Africa (53%), Mongolia (57%), India (60%) and higher survival rates in North America and Oceania (84-89%). Its study showed that differences in the 5-year cancer specific-survival were dependent on the country 9. After a comparison of the SEER database with an institutional Brazilian database adjusted by the same characteristics, a similar 10-year global survival rate was observed according to clinical stage, which reminds us to consider the influence of the percentage of early clinical stage cases on the overall survival, and the importance of long-term follow-up. In this publication, the cancer-specific survival was not discussed, and differences were observed in patients with clinical stage I (3.6 at 5-years and 13.0% at 10-years; *p*=0.001) and clinical stage II (4.7 pp at 5-years and 6.6 pp at 10-years; *p*=0.001) disease. However, these differences were associated with the quality of mortality data, the loss of follow-up information, as well as differences in treatment protocols and molecular subtypes, which renders it difficult to make comparisons among countries 62.

If we consider that mammographic screening and early diagnosis are not related to an increase in the survival, as shown in some randomized studies, we must not forget that these studies were performed in developed countries, where favorable conditions are present for diagnosis and treatment. In reality, the opposite conditions are present in DCs, a fact that reflects the lower rate of early diagnosis and poor survival. It is therefore important to have a progressive structuring of public health systems. To evaluate this condition, we found possible indicators that can be reported and that can be used in future studies performed in DCs (Table 1).

The identification of subpopulations or analysis based on socioeconomic conditions helps to understand the context of a population that is more dependent on the public healthcare system. Similarly, the observation of temporal data allows us to evaluate progress relating to the structuring of the public healthcare system. Limitations in diagnosis and treatment lead to a high mortality/incidence ratio 74; specifically, diagnosis is delayed, and many treatments are not performed based on protocols 20.

When using the PICOTS methodology for diagnostic tests 11, the Timing (T) and Setting (S) are observed, but studies of these factors were not available. Therefore, we present general aspects (PICO; Table 1). Some review articles on the subject were included in the overall review, but they were not selected in the 45 articles that determined the indicators because they do not show potential indicators.

Two aspects should be emphasized in this study. In the initial evaluation, the search was conducted using keywords that are associated with case-control studies. This search revealed 4 publications, but none of these publications included possible indicators, which led us to review general articles to identify possible indicators. Furthermore, the separation between DCs and underdeveloped countries presented another problem. The literature evaluated lacks a clear separation between the two, despite the possibility of using World Bank classifications 90. Therefore, underdeveloped countries could not be excluded from the evaluation.

Limitations of the present study include the following: only 1 database was used; the evaluation of articles was not based on the level of evidence but rather on available data, which we attempted to systematically evaluate; and comparisons of all indicators in developed countries were not reported. The objective was to identify potential indicators identified by a systematic methodology. Consequently, indirect indicators that can be used in DCs were identified, which elucidated the conditions relating to breast cancer diagnosis in DCs. The systematic identification and description of these indicators will facilitate comparisons among countries, the evaluation of public services, and the evaluation of outcomes of the progressive structuring of healthcare systems.

Currently, the validity of mammographic screening and mammography is under discussion, but such discussions are carried out in countries with structured healthcare systems that allow for diagnosis and treatment, irrespective of who absorbs the costs of this process. A better understanding of the alternate reality, that is, comparisons between limited public healthcare systems in terms of technology or access to mammography, will enable us to better understand the benefits of this diagnostic modality and mammographic screening.

Mammographic screening is proved to be beneficial when it is performed in an organized and regular manner in the form of a national public health policy or when the per capita income of the population allows it to absorb most of the costs. Therefore, organized screening is difficult in DCs, but the progressive structuring of healthcare services can significantly attenuate this problem. The socioeconomic dependence and late diagnosis reflect the higher morbidity and mortality rates of breast cancer in DCs. The impact of mammographic screening may be more beneficial in DCs than what is observed in developed countries. This structuring can be evaluated using indicators relating to diagnostic quality or methodology, and this study identified these indicators. These indicators will facilitate the evaluation of the improvement in health systems related to breast cancer and will allow comparisons among countries. This will provide us with a better understanding of the real impact of mammographic screening in DCs.

## AUTHOR CONTRIBUTIONS

Vieira RA participated in the study design, article selection, data analysis and writing of the manuscript. Biller G participated in the article selection and data analysis. Uemura G participated in the data analysis and discussion. Ruiz CA participated in the data analysis and discussion. Curado MP participated in the study design and discussion. All authors read and approved the final written version of the manuscript.

## Figures and Tables

**Table 1:** Indirect indicators related to breast cancer diagnosis in DCs.

PICOT		Factor	Indicator
Population	Breast cancer	Epidemiological	Incidence of breast cancer
Intervention	Methodology or diagnostic condition	Diagnosis	Diagnostic strategy methodology National Guidelines; Screening age range Mammography infrastructure; % of population coverage % of mammographies performed Structuring of network with screening rounds
		Quality	Form of presentation of symptoms at diagnosis Time to diagnosis % of early-stage cases (CS 0, CS 0+1, localized/regional disease)
Comparator	Control		Trend curves/temporal data Vulnerable subpopulations Comparison among countries
Outcome	Final outcome	Survival	Mortality/incidence ratio Survival according to staging

CS = clinical stage.
